# Differential Effects of Low and High Radiation Dose Rates on Mouse Spermatogenesis

**DOI:** 10.3390/ijms222312834

**Published:** 2021-11-27

**Authors:** Min Ji Bae, Min Kook Kang, Yong Uk Kye, Jeong-Hwa Baek, Ye-Ji Sim, Hae-June Lee, Yeong-Rok Kang, Wol Soon Jo, Joong Sun Kim, Chang Geun Lee

**Affiliations:** 1 Research Center, Dongnam Institute of Radiological & Medical Sciences, Busan 619-953, Korea; wlalsqo@dirams.re.kr (M.J.B.); minkooking@dirams.re.kr (M.K.K.); kyu0610@dirams.re.kr (Y.U.K.); jihan918@dirams.re.kr (J.-H.B.); dbr1228@dirams.re.kr (Y.-J.S.); yeongrok@dirams.re.kr (Y.-R.K.); sailorjo@dirams.re.kr (W.S.J.); 2 Division of Radiation Biomedical Research, Korea Institute of Radiological & Medical Sciences, 75 Nowon-ro, Nowon-gu, Seoul 01812, Korea; hjlee@kirams.re.kr; 3 College of Veterinary Medicine and BK21 Plus Project Team, Chonnam National University, 77 Yongbong-ro, Buk-gu, Gwangju 61186, Korea

**Keywords:** ionizing radiation, low dose rate, spermatogenesis, testis

## Abstract

The adverse effects of radiation are proportional to the total dose and dose rate. We aimed to investigate the effects of radiation dose rate on different organs in mice. The mice were subjected to low dose rate (LDR, ~3.4 mGy/h) and high dose rate (HDR, ~51 Gy/h) radiation. LDR radiation caused severe tissue toxicity, as observed in the histological analysis of testis. It adversely influenced sperm production, including sperm count and motility, and induced greater sperm abnormalities. The expression of markers of early stage spermatogonial stem cells, such as Plzf, c-Kit, and Oct4, decreased significantly after LDR irradiation, compared to that following exposure of HDR radiation, in qPCR analysis. The compositional ratios of all stages of spermatogonia and meiotic cells, except round spermatid, were considerably reduced by LDR in FACS analysis. Therefore, LDR radiation caused more adverse testicular damage than that by HDR radiation, contrary to the response observed in other organs. Therefore, the dose rate of radiation may have differential effects, depending on the organ; it is necessary to evaluate the effect of radiation in terms of radiation dose, dose rate, organ type, and other conditions.

## 1. Introduction

The harmful effects of radiation doses above approximately 100 mGy have been well supported by a wealth of data [1]. However, the effects associated with radiation doses below 100 mGy are still debated because they are largely estimated based on the radiation studies on high doses and dose rates [2]. Low dose and low dose rate (LDR) are defined as less than 100 mGy and 0.1 mGy/min (averaged over one hour), respectively, for low-linear-energy-transfer (LET) radiations, such as X-rays and gamma rays, respectively, by the United Nations Scientific Committee on the Effects of Atomic Radiation (UNSCEAR) 2017 report. The DNA damage induced by relatively low-dose-rate radiation can predominantly be remedied by the cellular-DNA-damage repair activity; this results in reduced cell death compared to that induced by a high dose rate. However, it was also reported that cell death was enhanced in a certain range of LDR radiation, compared to high-dose-rate (HDR) radiation [3,4,5,6]. Thus, further investigation is required to evaluate the biological effects of low-dose and LDR radiation.

The testis, which produces sperm and testosterone, consists of seminiferous tubules, Sertoli, and Leydig cells. Sperms are produced during spermatogenesis. Stem cell spermatogonia, type A-single (A_s_), and produced paired (A_pr_) and aligned (A_al_) spermatogonia, which are further differentiated into A_1_, A_2_, A_3_, A_4_, intermediate (ln), and type-B spermatogonia. After the last mitotic division, type-B spermatogonia produced two primary spermatocytes that enter meiosis to generate haploid round spermatids. Through the differentiation process, the round spermatids produce elongated spermatids that are released as spermatozoa [7,8].

Spermatogenesis, which consists of several cell division and differentiation steps, is highly sensitive to radiation [9]. Upon exposure to radiation, DNA damage is induced in the spermatogonia and causes cell cycle arrest. If DNA damage is sufficiently high, the entire population of spermatogonia may die, resulting in infertility [10]. Among cell types in the process of spermatogenesis, A_1_~A_4_ spermatogonia which are actively proliferating are known to be more sensitive to radiation than the other cell types. Stem cell spermatogonia A_s_ are the most resistant to radiation [11,12,13]. Thus, the surviving stem cell spermatogonia, A_s_, after exposure to radiation, replenish their own population and also produce differentiated spermatogonia to initiate the recovery of spermatogenesis [11,14,15,16]. Delays between the radiation exposure and re-initiation of spermatogenesis may vary depending on the radiation dose and dose rate.

We investigated how the radiation dose rate affects various organs of mice. Histological examination, molecular analysis, and FACS were performed. Although a high radiation dose rate was associated with greater damage to various organs, we observed a contrasting pattern for the testis. The level of testicular damage was higher when exposed to a low-dose-rate radiation than when exposed to higher dose rates. To our knowledge, this is the first report showing that the radiation dose rate may have differential effects depending on the organ analyzed. Thus, extensive evaluation of the effect of radiation doses, dose rates, the effects on organs, and other condition is required.

## 2. Results

### 2.1. Low- and High-Dose-Rate Radiation Have Differential Effects on Mouse Organs

**Figure 1:**
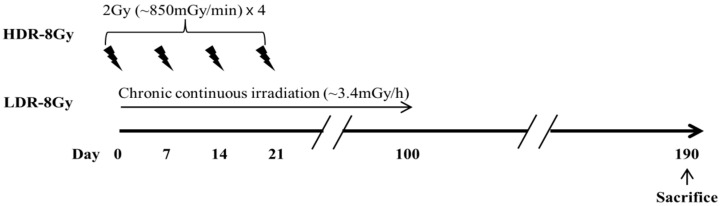
Schematic diagram of irradiation schedule, dose, and dose rate. Mice were irradiated with 8 Gy at high (HDR, ~850 mGy/min = ~51 Gy/h, 2 Gy × 4 times at one-week intervals) or low (LDR, ~3.4 mGy/h, continuously for ~100 days) dose rates, using a ^60^Co or ^137^Cs source, respectively. Radiation with high and low dose rates began at the same day.

**Figure 2:**
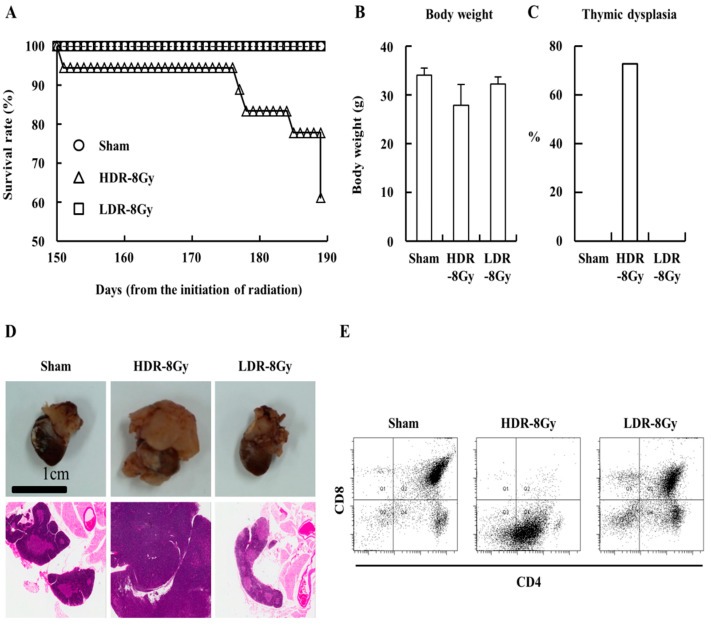
HDR-8 Gy (2 Gy x 4 times) induced thymic dysplasia, but not LDR-8 Gy. Mice were irradiated, as shown in Figure 1, and sacrificed for analysis at the indicated time point. (**A**) Survival rate of each group. (**B**) Average body weight of the mice for each group was shown as mean values + SD. (**C**) Thymic dysplasia incidence of each group. (**D**) Representative images of the thymus and histological examination of the thymus stained with H&E (magnification × 20). (**E**) Representative FACS analysis of the thymocytes of each group stained with anti-CD4 and CD8 antibodies.

To investigate how dose rate influences the radiation effects, mice were irradiated at LDR (~3.4 mGy/h) or HDR (~850 mGy/min, 2 Gy × 4 times at one-week intervals) for an equal dose (8 Gy) (Figure 1). Compared to the sham and LDR-8 Gy groups, the survival rate of the HDR-8 Gy group gradually decreased during the period following the end of radiation, despite no significant difference in body weight (Figure 2A,B). Mice were sacrificed and analyzed. One of the differences between the HDR-8 Gy and LDR-8 Gy groups was the induction of thymic dysplasia, which was determined by histological and FACS analyses (Figure 2C–E). It was reported that thymic dysplasia (lymphoma) was induced by the radiation protocol with 2 Gy × 4 times in one-week intervals [17,18,19].

Consistent with previous reports, ~70% of the HDR-8 Gy group developed thymic dysplasia. However, the LDR-8 Gy groups did not develop thymic dysplasia during the follow-up period after the end of irradiation (Figure 2C–E). Because the mice in both groups were exposed to the same dose (8 Gy), we hypothesized that the difference in thymic dysplasia induction may be associated with the difference in dose rate.

**Figure 3:**
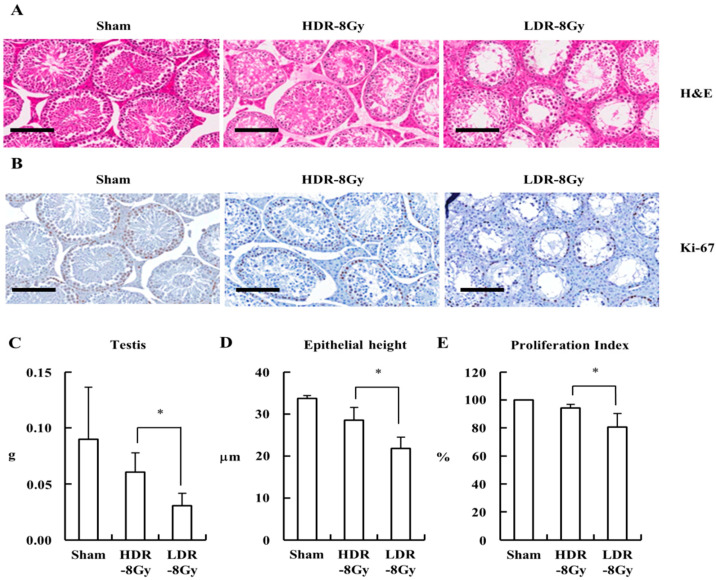
LDR-8 Gy induced more severe testicular toxicity than HDR-8 Gy. Mice were irradiated at a high or low dose rate, as shown in Figure 1, and sacrificed for analysis at the indicated time point. (**A**) Representative images (×400, Scale bar = 100 μm) of histological examination of the testis stained with H&E. (**B**) Representative images (×400, Scale bar = 100 μm) of histological examination of the testis stained with Ki7 antibody. (**C**) Average weight of testis, (**D**) epithelial height, and (**E**) proliferation index for each group were shown as mean values + SD. * A *p* < 0.05 between the compared groups.

Interestingly, the testes of the LDR-8 Gy group showed more weight loss and more severe tissue damage, which displayed an opposing trend to the thymic dysplasia incidence and survival rate (Figure 3A–E). Tissue damage was more severe and epithelial height was shorter in the LDR-8 Gy group than in the HDR-8 Gy group (Figure 3A,D). Proliferation was also further decreased in LDR-8 Gy than in HDR-8 Gy (Figure 3B,E). These were unexpected results, because the adverse effects were expected to be proportional to the total dose and dose rate [1,2].

**Figure 4:**
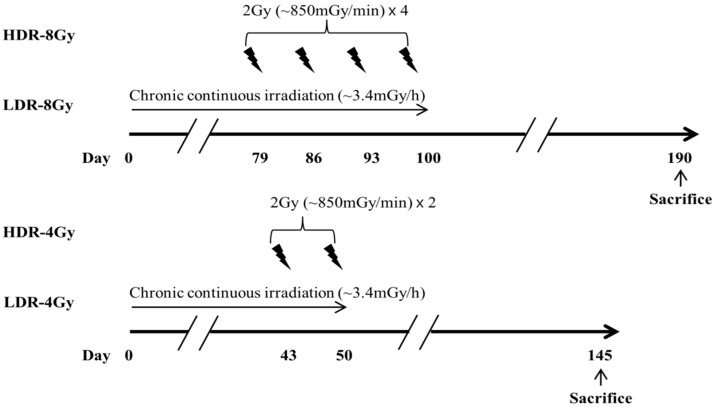
Schematic diagram of irradiation schedule, dose (4 and 8 Gy), and dose rate. Mice were irradiated with 4 or 8 Gy, either at high (HDR, ~850 mGy/min = ~51 Gy/h, 2 Gy × 2 or 4 times at one-week intervals) or low (LDR, ~3.4 mGy/h, continuously for ~50 or ~100 days) dose rates, using a ^60^Co or ^137^Cs source, respectively. Radiation with high and low dose rates ended at the same day.

**Figure 5:**
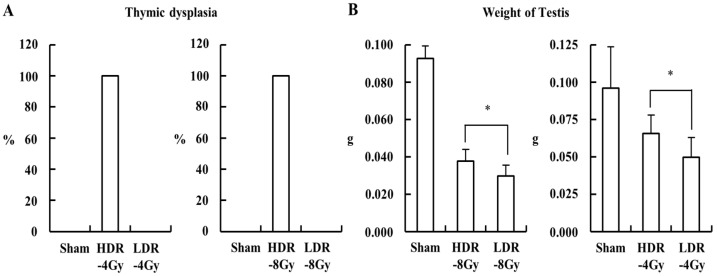
LDR (4 and 8 Gy) induced a more severe weight reduction of testis compared to HDR (4 and 8 Gy). Mice were irradiated at a high or low dose rate, as shown in Figure 4, and sacrificed for analysis at the indicated time point. (**A**) Thymic dysplasia incidence of each group. (**B**) Average weight of the testis for each group was shown as mean values + SD. * A *p* < 0.05 between the compared groups.

In the experiment, the HDR-8 Gy group had a longer recovery time (~170 days) after the end of radiation than the LDR-8 Gy group (~100 days) (Figure 1). To investigate whether the difference in the recovery time may be associated with the differences in the testicular toxicity between HDR-8 Gy and LDR-8 Gy, we modified the radiation and recovery schedule for the HDR and LDR groups, such that both groups have the same recovery time after the end of radiation (Figure 4). For the experiments with total radiation doses of 4 and 8 Gy, a higher incidence of thymic dysplasia was observed in the HDR-4 and -8 Gy groups (Figure 5A). However, the LDR group showed more weight loss in the testes than the HDR group (Figure 5B). Thus, the differences in testicular toxicity between the HDR and LDR groups may not be associated with the difference in recovery time.

### 2.2. LDR Radiation Induced More Defects in Sperm Production than HDR Radiation

**Figure 6:**
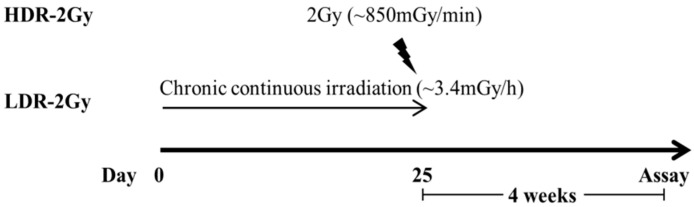
Schematic diagram of irradiation schedule, dose (2 Gy), and dose rate. Mice were irradiated with 2 Gy, either at high (HDR, ~850 mGy/min = ~51 Gy/h) or low (LDR, ~3.4 mGy/h) dose rate, with a ^60^Co or ^137^Cs source continuously for ~25 days, respectively. Radiation with high and low-dose rate ended at the same day.

**Figure 7:**
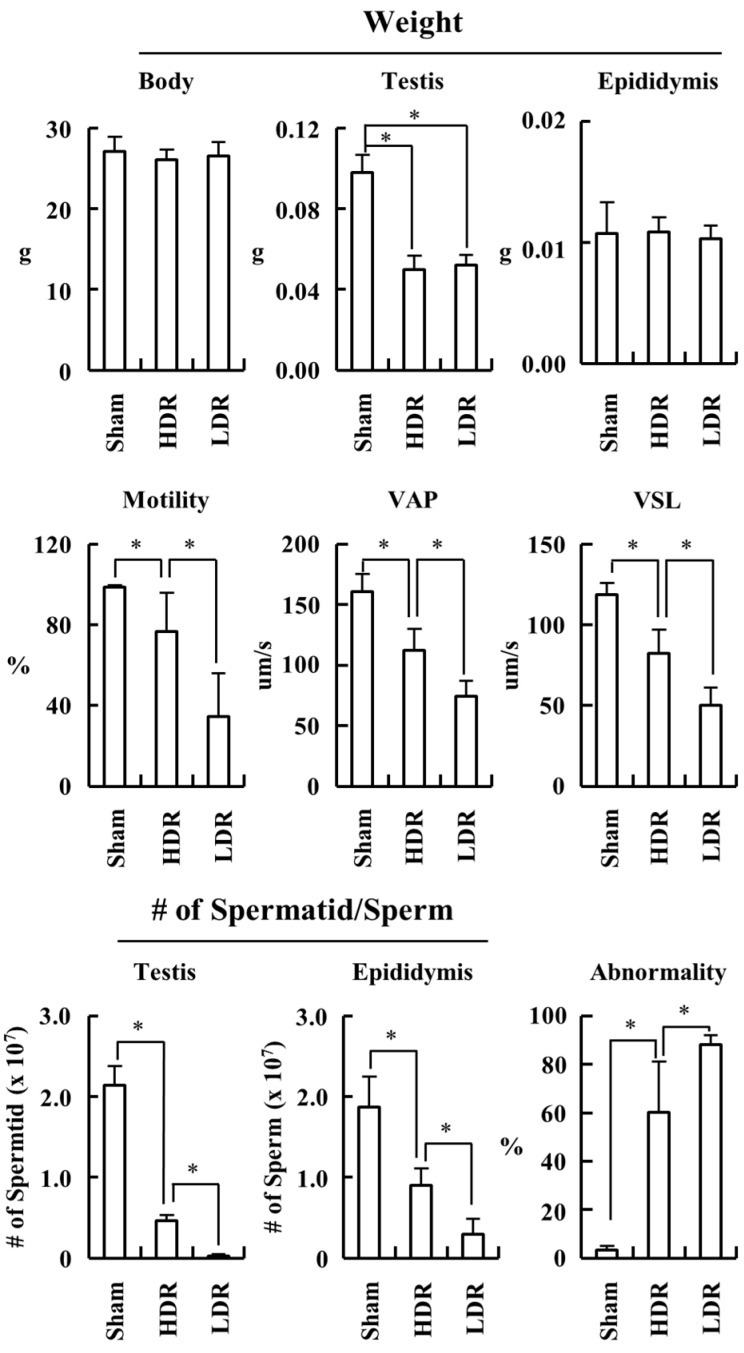
LDR induced more severe abnormalities and reduced sperm motility and counts than HDR. Mice were irradiated at high or low dose rates, as shown in Figure 6, and sacrificed for analysis at the indicated time point. Weight of the body, testis, and epididymis of each group was compared. Sperm motility, abnormality, and the counts of spermatid/sperm were analyzed, as described in Material and Methods. Data are shown as mean values + SD. VAP, path velocity (the average velocity of the smoothed cell path); VSL, straight-line velocity (the average velocity measured in a straight line from the beginning to the end of the track). * A *p* < 0.05 for the compared groups.

We investigated whether the same pattern of the dose-rate effect was observed during sperm production in the testis. Mice were irradiated with a total radiation dose of 2 Gy at low or high dose rates (Figure 6). Four weeks after the end of radiation, the testes of the two irradiated groups (HDR-2 Gy and LDR-2 Gy) were smaller than those of the sham group. However, there was no difference in the weight of the body, testis, and epididymis between the HDR-2 Gy and LDR-2 Gy groups, as is inconsistent with the results of 4 and 8 Gy experiments (Figure 7). This discrepancy may be due to the difference in the radiation doses and time interval after the end of radiation. Thus, we further investigated whether there was any difference in sperm count, motility, and abnormality between the HDR-2 Gy and LDR-2 Gy groups (Figure 7). The LDR-2 Gy group showed statistically decreased motility and total sperm/spermatid counts and increased abnormalities compared to the HDR-2 Gy group. Consistent with the toxicity results of the 4 and 8 Gy experiments, low-dose-rate radiation induced more adverse effects on spermatogenesis than high-dose-rate radiation. We further analyzed the types of abnormal sperms. However, there was no difference in the types of abnormalities between HDR-2 Gy and LDR-2 Gy; the predominant abnormalities were amorphous heads and detached tails (Table 1). This suggests that there may be no difference in the mechanism through which LDR and HDR induce adverse effects on spermatogenesis.

### 2.3. LDR Radiation Significantly Reduced Spermatogonia Cells Compared to HDR Radiation

**Figure 8:**
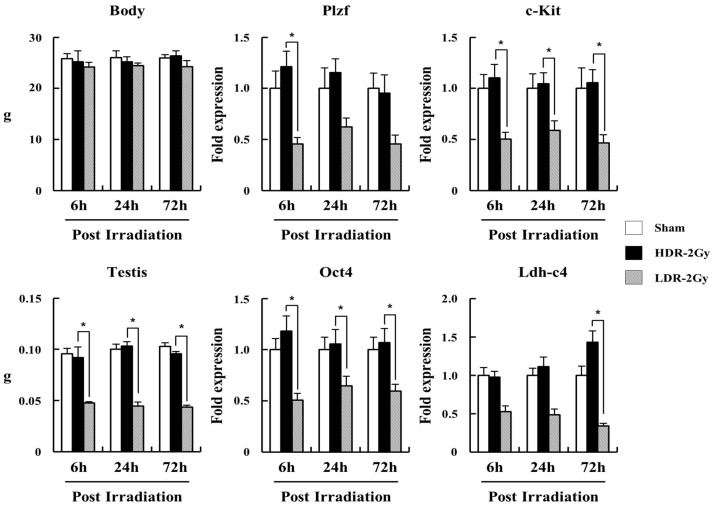
LDR reduced the population of spermatogonia cells further than HDR. Mice were irradiated at a high or low dose rate, as shown in Figure 6; and 6, 24, and 72 h after the end of irradiation, mice were sacrificed, and testicular single-cell suspension was obtained for qRT-PCR analysis, using the specific primers described in Materials and Methods. Data are shown as mean values + SD. * A *p* < 0.05 between the compared groups.

**Figure 9:**
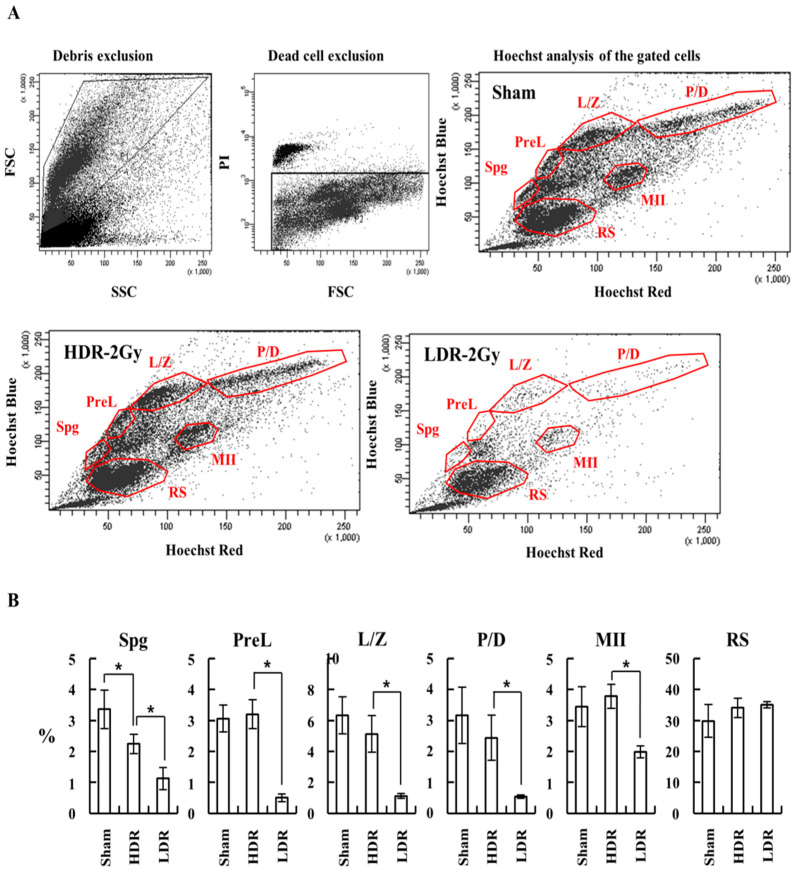
LDR group showed a more significant reduction in the population of spermatogonia and spermatocytes during meiosis than the HDR group. Mice were irradiated at high or low dose rate, as shown in Figure 6, and 24 h after the end of radiation, mice were sacrificed, and testicular single-cell suspension was obtained for FACS analysis, using Hoechst 33342 staining, as described in Materials and Methods. (**A**) Analysis of testicular single cell suspension by Hoechst 33342 staining. Debris was excluded based on light-scattering parameters, and dead cells were further excluded based on PI staining. The gated live cells were analyzed in a Hoechst Blue/Hoechst Red contour plot. Representative plots are shown. Spg, spermatogonia; PreL, preleptotene spermatocytes; L/Z, leptotene/zygotene spermatocytes; P/D, pachytene/diplotene spermatocytes; MII, meiosis II spermatocytes; RS, round spermatids. (**B**) Analysis of the population of spermatogonia, spermatocytes, and round spermatid. Data are shown as mean values + SD. * A *p* < 0.05 between the compared groups.

We investigated how LDR and HDR radiation differentially affect spermatogenesis. We performed a qRT-PCR analysis of mRNAs from testicular single cells, using specific primers for Plzf and Oct4 (undifferentiated spermatogonia), C-Kit (differentiating spermatogonia), and Ldh-c4 (preleptotene spermatocytes) [20,21,22]. Up to 72 h after the end of irradiation, all the markers for undifferentiated and differentiating spermatogonia and preleptotene spermatocytes were more significantly decreased in the LDH-2 Gy group than in the sham and HDR-2 Gy groups (Figure 8). Consistently, the weight of the testis in the LDR-2 Gy group was more decreased than that in the sham and HDR-2 Gy groups. To confirm the reduction in spermatogonia cells due to LDR radiation, testicular single cells were stained with Hoechst 33342 and analyzed for spermatogenic cell populations. Consistent with the results of the qRT-PCR analysis, the LDR radiation decreased the population of spermatogonia and spermatocytes during meiosis (Spg, PreL, L/Z, P/D, and MII), more significantly than the HDR radiation (Figure 9A,B). The population of round spermatids (RSs) was not significantly affected by LDR radiation. It takes approximately 20 days for primary spermatocytes to differentiate into RS. The RS population at the time point may not be exposed to radiation for enough time, because they initiate differentiation from spermatogonia stem cells before or during the early period of the irradiation (~25 days for 2 Gy at LDR). These results suggest that LDR chronic irradiation resulted in the reduction of spermatogonia cells in the early stages of spermatogenesis and cells during meiosis, possibly due to cell death from radiation-induced DNA damage. The reduction in the spermatogonia cell population after LDR radiation may reflect the reduction in viable sperm 4 weeks later. In addition, other surviving spermatogonia cells in the LDR group with unrepaired DNA damage may result in sperm with less motility and higher abnormality.

## 3. Discussion

Ionizing radiation induces DNA damage, which may result in cell death or mutations that lead to oncogenesis. In addition, radiation-induced DNA damage in the testis may be a cause of male infertility by inducing cell death in the spermatogonia cells and production of abnormal sperms [23]. Studies on the effect of radiation on male infertility and spermatogenesis have been extensively evaluated in terms of radiation doses [23,24,25,26]. However, the effects of LDR radiation (below 6 mGy/h, UNSCEAR 2017 report) on testis and spermatogenesis have not been studied as thoroughly. The increasing adverse effects have been proposed to be proportional to the dose rate for a given dose, suggesting that mutation frequency may increase as the dose rate is increased [1,2]. This phenomenon is known as the direct dose rate (DR) effect. However, it has been reported that there is an inverse DR effect below the 60~600 mGy/h range [27]. Thus, the mutant frequency due to radiation at 6 mGy/h (the upper limit of LDR, UNSCEAR 2017 report) may be higher than at the 60~600 mGy/h dose rate. It is believed that the activation of DNA repair below a certain range of dose rates is diminished, possibly due to a low ratio of radiation-induced DNA damage (signal) to spontaneous background DNA damage (noise) [27]. This could potentially explain why chronic LDR irradiation induced more adverse effects on testis and spermatogenesis than HDR irradiation. In our experiment, DNA damage in the spermatogonia cells and cells during meiosis induced by chronic LDR irradiation (~3.4 mGy/h) may not be efficiently repaired, and the accumulated mutations may result in enhanced cell death compared to those induced by HDR radiation.

The inverse DR effect may explain the differences in the testicular damages induced by LDR and HDR radiation. However, HDR radiation induced more adverse effects on the thymus and other organs besides the testis, as compared to LDR radiation (Figure 2 and data not shown). We believe that this discrepancy may be due to differences in the cell-proliferation rates between organs. Previous studies on the inverse DR effect have been performed by using an in vitro culture system for somatic mutations and spermatogonia cells for germline mutations [27]. Cells in both the systems proliferated efficiently. Although LDR induces mutations at a slower rate, which may permit cellular-DNA-damage repair mechanisms to function, cells with mutations may undergo cell death during subsequent cell-cycle stages. However, somatic cells in mice may not proliferate as efficiently as cells in in vitro cell culture systems. In mice, most somatic cells with LDR radiation-induced mutations may not enter the next cell cycle and persist at the G1 phase without cell death. Thus, we may not observe an inverse DR effect in the somatic cells of mice irradiated at LDR.

Another factor to be considered for the difference in testicular toxicity between LDR and HDR is the difference in radiation time between the LDR and HDR groups. Radiation was delivered in a relatively shorter time in the HDR group. Thus, early spermatogonia cells, such as type A-single (A_s_), may repair DNA damage and then resume spermatogenesis, hence having a higher chance of producing normal sperm. However, chronic LDR irradiation may induce DNA damage in early spermatogonia cells during cell division, which is important to replenish downstream spermatogonia cells, resulting in cell death or mutation in more differentiated spermatogonia cells.

Our previous study showed that chronic LDR radiation (dose, 1.7~2 Gy; dose rate, ~3.5 mGy/h) induced significant testicular and sperm damage accompanied by increasing permeability of the blood–testis barrier and decreasing the gene expression of DNMT1 and HDAC1 [28,29]. Downregulation of ZO (Zonula occludens)-1 and occludin-1 expression by LDR radiation was associated with the increased permeability of the blood–testis barrier, which plays a role in the apoptosis or necrosis of spermatogenic cells [30]. Besides DNA damage, these changes in permeability of the blood–testis barrier and gene expression of DNMT1 and HDAC1 by LDR chronic radiation may influence the spermatogenic cells, resulting in enhanced tissue damage and reduced sperm production.

Shin et al. reported that fewer abnormal forms of sperm were induced in LDR (0.7 mGy/h) irradiated mice than in those irradiated with a higher dose rate (0.8 mGy/min) [31]. These results appear to be inconsistent with our results, where reduced motility and higher abnormality were observed in mice irradiated with LDR (3.4 mGy/h) than in mice irradiated with HDR (0.85 mGy/h). We think that this discrepancy between two experiments may be due to the difference in LDRs used for the two experiments (0.7 vs. 3.4 mGy/h). Radiation at 0.7 mGy/h or lower may not induce DNA damage that is harmful enough to induce cell death in spermatogonia cells. One of possible explanations may be that there can be a threshold between 0.7 and 3.4 mGy/h for the induction of DNA damage that is sufficiently harmful to spermatogonia cells. The hypothesis may be investigated further if sensitive methods are available to detect very low levels of DNA damage in spermatogonia cells or other types of cells.

In our experiments, we have shown that the DR effect of radiation may result in different phenotypes depending on the organ. Several factors, including dose rate and proliferation rate of cell organs, can influence this phenomenon. Thus, we may need to evaluate the DR effects of radiation in vivo for various dose rates and organs in detail. The data from the systematic evaluation of the DR effect may be helpful to understand the responses of our bodies to ionizing radiation.

## 4. Materials and Methods

### 4.1. Animals

Balb/c male mice (4–5 weeks old) were purchased from the Central Lab. Animal Inc. (Seoul, Republic of Korea) and kept in a specific pathogen-free facility (temperature, 23 ± 2 °C; relative humidity, 50 ± 5%; artificial lighting, 8 a.m.–8 p.m.; air circulation, 13~18/hour; and feeding, a standard animal diet) at the Dongnam Institute of Radiological & Medical Sciences (DIRAMS). All protocols for animal experiments were approved by the Institutional Animal Care and Use Committee of DIRAMS (DIRAMS AEC-2017-005, 20 January 2017, AEC-2019-002, 9 January 2019, AEC-2020-003, 5 February 2020, and AEC-2021-001, 14 January 2021).

### 4.2. Irradiation

Mice (7~20 head/group) were exposed to radiation either at LDR (~3.4 mGy/h) or HDR (~51 Gy/h), with a ^137^Cs or ^60^Co source, respectively.

### 4.3. Evaluation of Radiation-Induced Toxicity by Histological Examination

Mice were sacrificed at the indicated time points after irradiation, and the body and organ weights were measured. The obtained organ samples were either fixed in the Bouin’s fluid solution or histological analysis or frozen in liquid nitrogen and stored in a −80 °C deep freezer for biological analysis. Histological analysis was performed according to previously described protocols [28,29]. Briefly, the fixed organs were embedded in paraffin, and sections of the organs were made, stained for hematoxylin and eosin (H&E) or for Ki-67, and analyzed under a microscope. The epithelial height of the seminiferous tubules was measured by using testis sections, as described previously [28,32]. For the proliferation index analysis, the proliferating activity of the testis sections was measured by immunohistochemistry of Ki-67 antibody (DRM004, 1:200, Acris Antibodies GmbH, Herford, Germany), as described previously [32].

### 4.4. Fluorescence-Activated Cell Sorting (FACS) Analysis of Thymocytes

The thymuses were disaggregated by pressing through a 40 µm cell strainer (Fisher Scientific, Cat# 08-771-1) to obtain a single cell suspension. Thymocytes were stained with PerCP-Cy5.5-anti-CD3, APC-anti-CD4, and FITC-anti-CD8 antibodies (BD Bioscience, Franklin Lakes, NJ, USA) and analyzed by using FACS Aria (Becton, Dickinson and Company, Franklin Lakes, NJ, USA), using FACSDiva software v6.1.3 (BD Biosciences, Franklin Lakes, NJ, USA).

### 4.5. Examination of Sperm Motility, Count, and Abnormality

After weighing the left testis and caudal epididymis of all males at termination, the sperm number in the testis and epididymis, sperm motility, and sperm morphology were evaluated by following a previously described protocol with minor modification [33,34,35]. Sperm motility and morphology analyses were conducted by using sperm collected from the vas deferens region of the epididymis. Sperm motility, path velocity (VAP), and straight-line velocity (VSL) were analyzed and calculated by using the HTM-TOX IVOS system for sperm analysis (Version 12.3, Hamilton-Thorne Research, Beverly, MA, USA) (Supplementary Materials). The smears made with the collected sperm were fixed in methanol and stained with 10% Giemsa in Sorensen buffer. A total of 200 sperms/animal were scored for sperm morphology under a microscope. Abnormal shape was subcategorized as follows: small head, amorphous head, two heads, excessive hook, blunt hook, folded tail, short tail, two tails, and detached tail.

### 4.6. Purification of Testicular Single Cells

Testicular single-cell suspensions comprising most of the cells that are present during spermatogenesis were obtained by following a previously described protocol, with minor modifications [36]. Briefly, the tunica albuginea was removed, and the testes were incubated in 200 U/mL collagenase type I and 5 μg/mL DNase I in GBSS, with shaking (150 rpm for 10 min), at 35 °C. The testes were dispersed by using a transfer pipette, and the supernatant containing interstitial testicular cells was removed. After incubation with GBSS containing 200 U/mL collagenase type I and 5 μg/mL DNase I and 0.025% (*w*/*v*) trypsin for 25 min under the same conditions as in the previous step, the enzymatic reaction was stopped by the addition of fetal bovine serum up to 10%, and then the cell suspension was passed through a 100 μm nylon cell strainer. A testicular single-cell suspension was obtained by centrifugation.

### 4.7. FACS Analysis of Spermatogenesis Stages

The testicular single-cell suspension was stained with Hoechst 33342 (Life Technologies, Cat# H3570, Carlsbad, CA, USA) by following a previously described protocol with minor modifications and subjected to analysis on FACS Aria (Becton, Dickinson and Company), using the FACSDiva software v6.1.3 (BD Biosciences, Franklin Lakes, NJ, USA) [36]. Hoechst was excited by using a UV (375 nm) laser, and the wide emission spectrum of the dye was detected in the “Hoechst Blue” (450/50 nm band-pass filter) and the “Hoechst Red” (670/50 nm band-pass filter). The latter was also used to detect PI. A dichroic mirror (635 nm long pass filter) was used to split the emission wavelengths.

### 4.8. qRT-PCR Analysis for Spermatogenesis Stage-Specific Markers

Total cellular RNA was extracted from testicular single cells, using the RNeasy Mini kit (Qiagen, Valencia, CA, USA), according to the manufacturer’s instructions, and cDNA was synthesized by using an iScript cDNA synthesis kit (Bio-Rad, Hercules, CA, USA). Then qRT PCR was performed by using cDNA and SYBR Green qPCR master mix (Enzynomics, Daejeon, R.O.K.), with the specific primers shown below, using the CFX96 Real-Time System (Bio-Rad, Hercules, CA, USA). The qPCR reaction parameters were 5 min at 95 °C, 40 cycles of 10 s at 95 °C, 10 s at 55 °C, and 10 s at 70 °C. A melting curve step (55 to 95 °C, at increments of 0.5 °C) was followed at the end of the qPCR. The primers used were as follows: GAPDH (Forward: 5′-GGGTGAGGCCGGTGCTGAGT-3′, Reverse: 5′-TGACCCGTTTGGCTCCACCCT-3′), c-Kit (Forward: 5′- TACAGGAGCAGAGCAAAGGTG-3′, Reverse: 5′-GCCTGGATTTGCTCTTTGTTGT-3′), Ldh-c4 (Forward: 5′-CAGTTATAAACTCGCCAC-3′, Reverse: 5′-ATTGATCTTAACAGGTTTCC-3′), Oct4 (Forward: 5′-TCAGACTTCGCCTTCTCACC-3′, Reverse: 5′-AGTTGCTTTCCACTCGTGCT-3′), and Plzf (Forward: 5′-CCTCACCAACCTTTCTT-3′, Reverse: 5′-ATCTTTGTCAGATCCATGA-3′).

### 4.9. Statistical Analysis

Results are expressed as the mean + SD. To determine statistical significance, the data were analyzed by using GraphPad Prism (Ver. 8.4.3) for Window (GraphPad Software, San Diego, CA, USA). ANOVA with Tukey’s post hoc test for multiple comparisons was performed to determine significant differences between more than two groups. A *p* < 0.05 was considered statistically significant.

## Figures and Tables

**Table 1:** Percentage of abnormal forms of sperms in the epididymis of the control and the irradiated mice.

%		Small Head	Amorphous Head	Two Head	Excessive Hook	Blunt Hook	Folded Tail	Short Tail	Two Tail	Detached Tail
**Sham**		0	0	0	0	0	0.1 ± 0.2	0	0	3.1 ± 1.9
**2 Gy**	**HDR**	0.1 ± 0.2	9.3 ± 6.3	0	0	0	4.6 ± 2.4	0.7 ± 1.0	0.3 ± 0.6	45.1 ± 12.7
	**LDR**	0.4 ± 0.4	12.8 ± 4.7	0	0	0	2.6 ± 1.2	1.3 ± 0.7	1.2 ± 2.2	70.0 ± 3.6

200 sperms/head (*n* = 7 head/group) were evaluated.

## Data Availability

The data that support the findings of this study are available from the corresponding authors upon reasonable request.

## Supplementary Materials

The following are available online at https://www.mdpi.com/article/10.3390/ijms222312834/s1.

## Abbreviations

qRT-PCR: real-time quantitative reverse transcription; FACS, fluorescence-activated cell sorting; LD, low dose; LDR, low dose rate; HDR, high dose rate; UNSCEAR, United Nations Scientific Committee on the Effects of Atomic Radiation; H&E, hematoxylin and eosin; VAP, path velocity; VSL, straight line velocity; GBSS, Gey’s balanced salt solution; SD, standard deviation; ANOVA, one-way analysis of variance.

## References

[1] Hall E.J. (1991). Weiss lecture. The dose-rate factor in radiation biology. Int. J. Radiat. Biol..

[2] Rühm W., Woloschak G., Shore R.E., Azizova T., Grosche B., Niwa O., Akiba S., Ono T., Suzuki K., Iwasaki T. (2015). Dose and dose-rate effects of ionizing radiation: A discussion in the light of radiological protection. Radiat. Environ. Biophys..

[3] Amundson S.A., Chen D.J. (1996). Inverse dose-rate effect for mutation induction by gamma-rays in human lymphoblasts. Int. J. Radiat. Biol..

[4] Matsuya Y., Tsutsumi K., Sasaki K., Date H. (2015). Evaluation of the cell survival curve under radiation exposure based on the kinetics of lesions in relation to dose-delivery time. J. Radiat. Res..

[5] Mitchell C.R., Folkard M., Joiner M.C. (2002). Effects of exposure to low-dose-rate (60)co gamma rays on human tumor cells in vitro. Radiat. Res..

[6] Stevens D.L., Bradley S., Goodhead D.T., Hill M.A. (2014). The Influence of Dose Rate on the Induction of Chromosome Aberrations and Gene Mutation after Exposure of Plateau Phase V79-4 Cells with High-LET Alpha Particles. Radiat. Res..

[7] Kanatsu-Shinohara M., Shinohara T. (2013). Spermatogonial Stem Cell Self-Renewal and Development. Annu. Rev. Cell Dev. Biol..

[8] Phillips B.T., Gassei K., Orwig K.E. (2010). Spermatogonial stem cell regulation and spermatogenesis. Philos. Trans. R. Soc. B Biol. Sci..

[9] Kovacs G.T., Stern K. (1999). Reproductive aspects of cancer treatment: An update. Med. J. Aust..

[10] De Rooij D.G., Russell L.D. (2000). All you wanted to know about spermatogonia but were afraid to ask. J. Androl..

[11] Meistrich M.L., Wilson G., Mathur K., Fuller L.M., Rodriguez M.A., McLaughlin P., Romaguera J.E., Cabanillas F.F., Ha C.S., Lipshultz L.I. (1997). Rapid recovery of spermatogenesis after mitoxantrone, vincristine, vinblastine, and prednisone chemotherapy for Hodgkin’s disease. J. Clin. Oncol..

[12] Van Der Meer Y., Huiskamp R., Davids J.A., Van Der Tweel I., De Rooij D.G. (1992). The sensitivity of quiescent and proliferating mouse spermatogonial stem cells to X irradiation. Radiat. Res..

[13] Van Der Meer Y., Huiskamp R., Davids J.A., Van Der Tweel I., De Rooij D.G. (1992). The sensitivity to X rays of mouse spermatogonia that are committed to differentiate and of differentiating spermatogonia. Radiat. Res..

[14] Meistrich M.L., Trostle P.K., Frapart M., Erickson R.P. (1977). Biosynthesis and localization of lactate dehydrogenase X in pachytene spermatocytes and spermatids of mouse testes. Dev. Biol..

[15] Mian T.A., Suzuki N., Glenn H.J., Haynie T.P., Meistrich M.L. (1977). Radiation damage to mouse testis cells from [99mTc] pertechnetate. J. Nucl. Med..

[16] Van Beek M.E.A.B., Meistrich M.L., De Rooij D.G. (1990). Probability of self-renewing divisions of spermatogonial stem cells in colonies, formed after fission neutron irradiation. Cell Prolif..

[17] Boniver J., Humblet C., Rongy A.M., Delvenne C., Delvenne P., Greimers R., Thiry A., Courtoy R., Defresne M.P. (1990). Cellular aspects of the pathogenesis of radiation--induced thymic lymphomas in C57 BL mice (review). Vivo.

[18] Humblet C., Greimers R., Boniver J., Defrense M.P. (1997). Stages in the development of radiation-induced thymic lymphomas in C57 BL/Ka mice: Preleukemic cells become progressively resistant to the tumor preventing effects of a bone marrow graft. Exp. Hematol..

[19] Muto M., Sado T., Hayata I., Nagasawa F., Kamisaku H., Kubo E. (1983). Reconfirmation of indirect induction of radiogenic lymphomas using thymectomized, irradiated B10 mice grafted with neonatal thymuses from Thy 1 congenic donors. Cancer Res..

[20] Barrios F., Filipponi D., Campolo F., Gori M., Bramucci F., Pellegrini M., Ottolenghi S., Rossi P., Jannini E.A., Dolci S. (2012). SOHLH1 and SOHLH2 control Kit expression during postnatal male germ cell development. J. Cell Sci..

[21] Filipponi D., Hobbs R.M., Ottolenghi S., Rossi P., Jannini E.A., Pandolfi P.P., Dolci S. (2007). Repression of kit Expression by Plzf in Germ Cells. Mol. Cell. Biol..

[22] Goldberg E., Eddy E.M., Duan C., Odet F. (2009). LDHC: The Ultimate Testis-Specific Gene. J. Androl..

[23] Fukunaga H., Butterworth K., Yokoya A., Ogawa T., Prise K.M. (2017). Low-dose radiation-induced risk in spermatogenesis. Int. J. Radiat. Biol..

[24] Grewenig A., Schuler N., Rübe C.E. (2015). Persistent DNA Damage in Spermatogonial Stem Cells After Fractionated Low-Dose Irradiation of Testicular Tissue. Int. J. Radiat. Oncol..

[25] Qi L., Li J., Le W., Zhang J. (2019). Low-dose ionizing irradiation triggers apoptosis of undifferentiated spermatogonia in vivo and in vitro. Transl. Androl. Urol..

[26] Wdowiak A., Skrzypek M., Stec M., Panasiuk L. (2019). Effect of ionizing radiation on the male reproductive system. Ann. Agric. Environ. Med..

[27] Vilenchik M.M., Knudson A.G. (2000). Inverse radiation dose-rate effects on somatic and germ-line mutations and DNA damage rates. Proc. Natl. Acad. Sci. USA.

[28] Gong E.J., Shin I.S., Son T.G., Yang K., Heo K., Kim J.S. (2014). Low-dose-rate radiation exposure leads to testicular damage with decreases in DNMT1 and HDAC1 in the murine testis. J. Radiat. Res..

[29] Son Y., Heo K., Bae M.J., Lee C.G., Cho W.S., Kim S.D., Yang K., Shin I.S., Lee M.Y., Kim J.S. (2015). Injury to the blood-testis barrier after low-dose-rate chronic radiation exposure in mice. Radiat. Prot. Dosim..

[30] Hou W.-G., Zhao J., Li Z., Li W., Li T., Xiong L.-Z., Zhang Y.-Q. (2012). Effects of Electromagnetic Pulse Irradiation on the Mouse Blood-testicle Barrier. Urology.

[31] Shin S.C., Kang Y.M., Jin Y.W., Kim H.S. (2009). Relative morphological abnormalities of sperm in the caudal epididymis of high- and low-dose-rate gamma-irradiated ICR mice. J. Radiat. Res..

[32] Lee W., Son Y., Jang H., Bae M.J., Kim J., Kang D., Kim J.S. (2015). Protective Effect of Administered Rolipram against Radiation-Induced Testicular Injury in Mice. World J. Men’s Health.

[33] Carvajal G., Brukman N.G., Muñoz M.W., Battistone M.A., Guazzone V.A., Ikawa M., Haruhiko M., Lustig L., Breton S., Cuasnicu P.S. (2018). Impaired male fertility and abnormal epididymal epithelium differentiation in mice lacking CRISP1 and CRISP4. Sci. Rep..

[34] Harris T., Marquez B., Suarez S., Schimenti J. (2007). Sperm Motility Defects and Infertility in Male Mice with a Mutation in Nsun7, a Member of the Sun Domain-Containing Family of Putative RNA Methyltransferases1. Biol. Reprod..

[35] Rasgele P.G. (2014). Abnormal sperm morphology in mouse germ cells after short-term exposures to acetamiprid, propineb, and their mixture. Arch. Ind. Hyg. Toxicol..

[36] Gaysinskaya V., Soh I.Y., van der Heijden G.W., Bortvin A. (2014). Optimized flow cytometry isolation of murine spermatocytes. Cytom. Part A.

